# Leonurine Promotes the Osteoblast Differentiation of Rat BMSCs by Activation of Autophagy via the PI3K/Akt/mTOR Pathway

**DOI:** 10.3389/fbioe.2021.615191

**Published:** 2021-02-23

**Authors:** Bingkun Zhao, Qian Peng, Enoch Hin Lok Poon, Fubo Chen, Rong Zhou, Guangwei Shang, Dan Wang, Yuanzhi Xu, Raorao Wang, Shengcai Qi

**Affiliations:** ^1^Department of Stomatology, Shanghai Tenth People’s Hospital, School of Medicine, Tongji University, Shanghai, China; ^2^Institute for Tissue Engineering and Regenerative Medicine, The Chinese University of Hong Kong, Hong Kong, China; ^3^School of Biomedical Sciences, The Chinese University of Hong Kong, Hong Kong, China

**Keywords:** BMSCs, leonurine, autophagy, osteoporosis, PI3K/Akt/mTOR pathway

## Abstract

**Background:**

Leonurine, a major bioactive component from *Herba leonuri*, has been shown to exhibit anti-inflammatory and antioxidant effects. The aim of this study was to investigate the effect of leonurine on bone marrow-derived mesenchymal stem cells (BMSCs) as a therapeutic approach for treating osteoporosis.

**Materials and Methods:**

Rat bone marrow-derived mesenchymal stem cells (rBMSCs) were isolated from 4-weeks-old Sprague–Dawley rats. The cytocompatibility of leonurine on rBMSCs was tested via CCK-8 assays and flow cytometric analyses. The effects of leonurine on rBMSC osteogenic differentiation were analyzed via ALP staining, Alizarin red staining, quantitative real-time polymerase chain reaction (qRT-PCR), and Western blot. Additionally, autophagy-related markers were examined via qRT-PCR and Western blot analyses of rBMSCs during osteogenic differentiation with leonurine and with or without 3-methyladenine (3-MA) as an autophagic inhibitor. Finally, the PI3K/Akt/mTOR signaling pathway was evaluated during rBMSC osteogenesis.

**Results:**

Leonurine at 2–100 μM promoted the proliferation of rBMSCs. ALP and Alizarin red staining results showed that 10 μM leonurine promoted rBMSC osteoblastic differentiation, which was consistent with the qRT-PCR and Western blot results. Compared with those of the control group, the mRNA and protein levels of Atg5, Atg7, and LC3 were upregulated in the rBMSCs upon leonurine treatment. Furthermore, leonurine rescued rBMSC autophagy after inhibition by 3-MA. Additionally, the PI3K/AKT/mTOR pathway was activated in rBMSCs upon leonurine treatment.

**Conclusion:**

Leonurine promotes the osteoblast differentiation of rBMSCs by activating autophagy, which depends on the PI3K/Akt/mTOR pathway. Our results suggest that leonurine may be a potential treatment for osteoporosis.

## Introduction

Osteoporosis is the most common bone disease and is found in one-tenth of the population aged above 50 years and one-quarter of the population aged above 80 years (Wright et al., 2014). Concurrently, the rate of occurrence of osteoporosis-related bone fractures is increasing; it is estimated that one in two women and one in five men aged above 50 years are at risk of bone fracture as a direct result of osteoporosis (Das and Crockett, 2013). This condition is a major clinical and public health concern, and high morbidity, disability, and mortality rates are associated with the disease. The risks of osteoporosis include age, inadequate calcium, vitamin D deficiency, female sex, lack of activity, smoking, alcohol consumption, and some chronic inflammatory diseases (Jing et al., 2016).

Osteoporosis is characterized by the fundamental imbalance between bone formation and resorption, which is caused by the overactivation of osteoclasts, an increase in cell apoptosis, and a decrease in the osteogenesis of BMSCs. This process results in a defining feature of osteoporosis: the loss of bone mass, which increases the risk of fracture. Inadequate bone formation by osteoblasts originating from bone marrow mesenchymal stem cells (BMSCs) to compensate for bone resorption by osteoclasts is considered the major cause of osteoporosis. Several studies have revealed that osteogenic differentiation is impaired in patients with osteoporosis and that recovering osteogenic capacities could treat osteoporosis (Jing et al., 2016).

Autophagy is a cellular process wherein the primary function is to clear damaged cellular components such as long-lived proteins and organelles, thus participating in conservation across multiple cell types. In pathological situations, autophagy plays an important role in maintaining bone homeostasis (Pierrefite-Carle et al., 2015). Current evidence indicates the dysregulation of autophagy as a major contributor to the development of metabolic disorders, including insulin resistance, diabetes mellitus, obesity, atherosclerosis, and osteoporosis. Autophagy plays an integral role in maintaining bone homeostasis, with substantial evidence demonstrating its contribution to the survival of osteoblasts, regulation of osteoblast differentiation, maintenance of bone mass, and improvement of bone strength (Yao et al., 2016; Geng et al., 2019). In contrast, decreased autophagy leads to an increase in BMSC apoptosis and inhibition of osteoblast differentiation (Nollet et al., 2014). Recent literature demonstrates the relationship between autophagy and osteoblast differentiation: BMSCs contain many autophagosomes in their initial differentiation stage, illustrating a fundamental linkage between autophagy and metabolism involved in osteoblastic differentiation (Nuschke et al., 2014). This evidence provides a logical foundation for pursuing the treatment of osteoporosis via autophagic regulation in BMSCs.

Current medical treatments for osteoporosis commonly encompass diphosphonate, calcitonin, estrogen receptor modulators, and parathyroid hormone (PTH); accordingly, such treatments can cause undesired side effects, including gastrointestinal responses, renal toxicity (Xu et al., 2013), hypocalcemia (Smith et al., 2009), and an increased risk of cancer (Choi et al., 2020). For example, bisphosphonates trigger osteoclast apoptosis with therapeutic efficiency reaching bottlenecks at 3–5 years post-treatment, along with an increased risk of atypical femoral fractures (Han et al., 2017); Denosumab was reported to be correlated with atypical femoral fracture and osteonecrosis of the jaw, reported drug withdrawal symptoms, an increase risk of bone fractures due to a rapid increase in bone remodeling, and potential cardiovascular events (Adhikary et al., 2018); PTH was reported to induce abnormal psychiatric symptoms and a restricted anabolic window within 2 years of treatment (Lee et al., 2010). Alternatively, traditional Chinese medicine has been used to prevent and treat osteoporosis with a recorded history of over a thousand years; the use of herbal medicine and its extracts, including *Epimedium*, *Salvia*, *Rehmanniae radix*, and *Ophiopognin*, has been shown to elicit fewer side effects and demonstrated higher sustainability over long-term periods than synthetic drugs (Guo et al., 2014; Xu et al., 2016; Liu et al., 2017; Xi et al., 2018). Despite a deficit in the current understanding of drug–receptor interactions and signaling pathways involved in herbal medicine, traditional Chinese medicine still holds potential and empirical evidence identifies it as a safe and effective alternative in treating osteoporosis.

Leonurine is a natural chemical compound extracted from the traditional Chinese herbal medicine *Herba leonuri*. In particular, its high anti-inflammatory and antioxidant effects have been extensively illustrated and can reduce the damage caused by excessive ROS in many diseases, such as cardiovascular diseases, ischemic stroke, and atherosclerosis (Liu et al., 2010; Loh et al., 2010; Zhang et al., 2012). In motor system-related diseases, leonurine could contribute to the reconstruction of cartilage in disease situations and alleviate osteoporotic progression in a rat model by inhibiting osteoclast differentiation (Hu et al., 2019; Chen et al., 2020). A recent study demonstrated that leonurine can regulate autophagy to ameliorate cognitive dysfunction (Liu et al., 2016). Therefore, we hypothesized that leonurine may be a selective medicine for targeting osteoporosis by regulating autophagic activity in BMSCs.

The aim of this study was to confirm the roles of leonurine in BMSCs and the underlying molecular mechanisms. The proliferation of BMSCs cocultured with leonurine was detected by CCK-8 assays and flow cytometry *in vitro*. The osteogenic differentiation of leonurine on BMSCs was evaluated by ALP staining, PCR, and Western blot (WB) analysis. The PI3K/Akt/mTOR pathway was analyzed using WBs.

## Materials and Methods

### Cell Culture and Differentiation Assays

Sprague–Dawley (SD) rat primary BMSCs were isolated from the bone marrow of 4-weeks-old rats. In brief, bone marrow cells were first flushed with cell culture medium and cultivated in Petri dishes. After 24 h of incubation, adherent cells (BMSCs) were extracted from each dish.

Extracted BMSCs were maintained in α-MEM (α-MEM, HyClone, United States) with 10% fetal bovine serum (FBS, Gibco, United States) and 1% penicillin/streptomycin (PS, Gibco, United States). The cells were cultured at 37°C/5% CO_2,_ and the medium was replaced every 3 days. With cells passaged upon reaching 80% confluency, BMSCs of passages 3–6 were used in the ensuing experiments.

For osteogenic differentiation, BMSCs were cultured with osteogenic induction medium containing 10% FBS, 1% penicillin/streptomycin, 50 μg/ml ascorbic acid (Sigma, United States), 10 mM sodium β-glycerophosphate (Sigma, United States), and 10 nM dexamethasone (Sigma, United States).

### The Biocompatibility of Leonurine on BMSCs

#### CCK8 (Cell Counting Kit-8) Assays

Cell proliferation was measured by Cell Counting Kit-8 assays (CCK-8, Dojindo, Shanghai, China) according to the manufacturer’s protocol with three replicates. The experimental group was treated with different concentrations of leonurine (0–100 μM). Briefly, the cells were cultivated in 96-well plates at a density of 3 × 10^3^ cells per well. The seeding density was measured after an initial overnight incubation with a CCK-8 assay to test whether they were equal. Another repeat was performed after 3 days of culture, in which the medium was replaced with 10 μl of CCK 8 solution dissolved in 200 μl of cell culture medium. The plate was incubated for 2 h before absorbance at 450 nm was measured.

#### Flow Cytometric Analysis of Cell Cycle and Apoptosis

BMSCs were seeded in six-well plates at a density of 2 × 10^4^ cells per well and treated with leonurine for 3 days. Subsequently, the cells were collected and fixed with 75% ethanol overnight at 4°C and washed with cold PBS twice before staining with 200 μl of PI/RNase staining buffer for 30 min at room temperature. Cell cycle distribution was detected by a BD FACSCanto II flow cytometer (BD BioScience, United States).

Apoptosis assays were further performed to ascertain the effect of leonurine on cell viability with FITC-Annexin V apoptosis detection kits (BD Bioscience, United States) according to the manufacturer’s protocols. Briefly, the cells were collected, washed with cold PBS, and resuspended in 1× binding buffer. Then, they were stained with 5 μl of Annexin V-FICT and 5 μM propidium iodide (PI) in the dark for 15 min at room temperature. Cell apoptosis was detected by a BD FACSCanto II flow cytometer (BD BioScience, United States).

### The Osteogenic Differentiation of BMSCs Induced by Leonurine

#### Alkaline Phosphatase (ALP) Staining and Alizarin Red Staining

BMSCs at a density of 2 × 10^4^ cells/well were seeded in 24-well plates. After 24 h, the baseline medium was replaced with osteogenic induction medium containing various concentrations of leonurine (0, 2, 5, and 10 μM). The cells were cultured in osteogenic induction medium for either 6 days (ALP staining) or 14 days (Alizarin red staining).

ALP staining was conducted to ascertain the effect of leonurine on BMSC differentiation. In brief, BMSCs were harvested after 6 days of culture and fixed with 4% paraformaldehyde for 10 min. An ALP color development kit (Beyotime, Shanghai, China) was used in the study according to the manufacturer’s protocols. Briefly, the cells were stained for 15 min and washed three times with PBS. The stained cells were subsequently observed under phase-contrast microscopy, with representative images captured.

Alizarin red staining was further performed to determine the degree of calcium deposition in BMSCs between the leonurine treatment groups at various concentrations. Briefly, BMSCs were harvested after 14 days of culture in osteogenic medium as outlined above. After fixation in 4% paraformaldehyde for 10 min, the cells were stained with Alizarin red staining kits (Beyotime, Shanghai) for 60 min and washed three times with ddH_2_O.

The stained cells were subsequently observed under phase-contrast microscopy, with representative images captured.

#### RNA Isolation and Quantitative Real-Time PCR (qRT-PCR) Analysis

BMSCs were seeded in six-well plates at a density of 5 × 10^4^ cells/well. After 24 h, osteogenic induction medium containing 10 μM leonurine was added to the experimental groups, with the control groups receiving medium without leonurine. Total RNA from the BMSCs was isolated following osteogenic induction for 6 days with the TRIzol (Invitrogen, United States) extraction method according to the manufacturer’s protocol. The total RNA concentration was measured by a Nanodrop system (Thermo Fisher Scientific, United States). cDNA was reverse transcribed with a PrimeScript RT reagent Kit (TaKaRa, Japan). qPCR was conducted with Hieff^TM^ qPCR SYBR^®^, Green Master Mix in an ABI 7500 Real-Time PCR System (Applied Biosystems, Foster City, CA, United States). Relative gene expression was calculated using the comparative 2^–Δ^ ^Δ^ ^*Ct*^ method. The primers used are listed in Table 1.

**TABLE 1:** Nucleotide sequences of primers used for qRT-PCR.

**Gene**		**Primer sequence(5′–3′)**
OCN	Forward primer	TGAGGACCCTCTCTCTGCTC
OCN	Reverse primer	GGGCTCCAAGTCCATTGTT
OPN	Forward primer	ATCTGAGTCCTTCACTG
OPN	Reverse primer	GGGATACTGTTCATCAGAAA
RUNX2	Forward primer	GCACCCAGCCCATAATAGA
RUNX2	Reverse primer	TTGGAGCAAGGAGAACCC
p62/SQSTM1	Forward primer	ACCCATCCACAGAGGCTGAT
p62/SQSTM1	Reverse primer	GCCTTCATCCGAGAAACCCA
ATG5	Forward primer	ACGTGTGGTTTGGACGGATT
ATG5	Reverse primer	AAGGCCGTTCAGTTGTGGTC
ATG7	Forward primer	TGGAGCATGCCTACGATGAC
ATG7	Reverse primer	TTTGGGGTCCATACATCCGC
GAPDH	Forward primer	CAGGGCTGCCTTCTCTTGT
GAPDH	Reverse primer	TCCCGTTGATGACCAGCTTC

#### Protein Extraction and WB Analysis

BMSCs at a density of 5 × 10^4^ cells/well were seeded in six-well plates and divided into a control group or treatment group (10 μM leonurine). Total protein from the rBMSCs was isolated following osteogenic induction for 6 days with RIPA buffer containing protease inhibitor and phosphatase inhibitor. Equal amounts of protein were separated and transferred onto nitrocellulose membranes (Millipore Corporation, Billerica, United States). Primary antibodies (GAPDH, OPG, and Runx2) were incubated with membranes at 4°C overnight, with the ensuing secondary antibody incubated at room temperature for 1 h. The membranes were visualized using the Odyssey LI-CDR system. GAPDH (1:2,000) was purchased from Cell Signaling Technology (CST, Beverly, MA, United States). OPG (1:500) and Runx2 (1:500) were purchased from Abcam (Cambridge, MA, United States).

### The Effect of Leonurine on Autophagy-Related Genes

BMSCs at a density of 5 × 10^4^ cells/well were seeded in six-well plates. For autophagic inhibition, BMSCs were treated with 2 mM 3-methyladenine (3-MA, Sigma, United States) with or without leonurine (10 μM). First, 3-MA was dissolved in dimethyl sulfoxide (DMSO) prior to addition to the culture medium, with the final concentration of DMSO below 0.1% of the medium. Alizarin red staining was performed for 14 days and ALP staining was performed for 6 days as described above.

Additionally, WB and qPCR were performed with the no treatment (control) group, leonurine treatment group, 3-MA treatment group, and 3-MA with leonurine treatment group. The antibodies LC3AB I/II (1:1,000) and ATG7 (1:500) used in WB analyses were purchased from Cell Signaling Technology (CST, Beverly, MA, United States); P62 (1:1,000) was purchased from Abcam (Cambridge, MA, United States). The primers used in the qPCR analysis are listed in Table 1.

### Analysis of Pathways Related to Leonurine

For PI3K activation, BMSCs were pretreated with 2 μM of the PI3K activator 740Y-P (APExBIO, United States) for 2 h separately prior to coculturing with 10 μM leonurine. Briefly, Y740Y-P was dissolved in DMSO, and the final concentration of DMSO contributed to less than 0.1% of the medium, inducing no notable cytotoxic effect. WB assays were performed as stated above. The experimental groups were the control group, pretreatment with the 740Y-P group, leonurine treatment with the 740Y-P pretreatment group, and the leonurine treatment group. Antibodies against AKT (1:1,000), p-AKT (1:1,000), and p-mTOR (1:1,000) were purchased from Cell Signaling Technology (CST, Beverly, MA, United States). Antibodies against PI3K (1:1,000) and p-PI3K (1:500) were purchased from Abcam (Cambridge, MA, United States).

### Statistical Analysis

Statistical analysis was performed by SPSS 20.0 (IBM, Somers, NY, United States). Each experiment was independently repeated at least three times. Differences between two groups were analyzed with unpaired Student’s *t*-test, and more than three groups were analyzed with one-way analysis of variance followed by the Bonferroni post-test. Data are presented as the mean ± standard error (SEM). *P* < 0.05 were considered to indicate significant differences.

## Results

### Leonurine Exhibits No Notable Toxicity and Contributes to BMSC Proliferation

**FIGURE 1:**
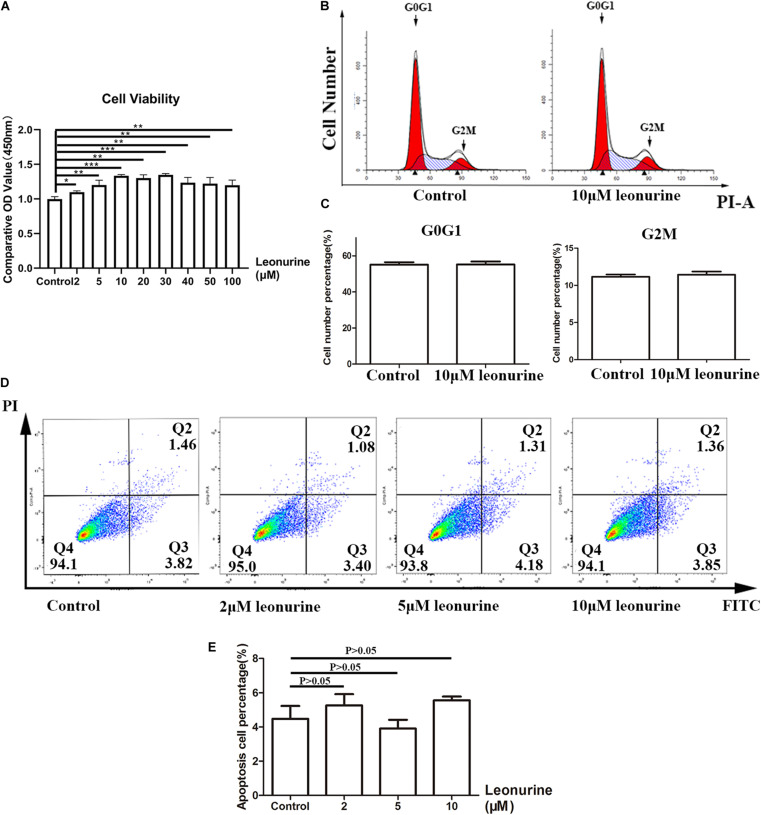
Assessment of toxicity of leonurine and its effect on BMSC proliferation. **(A)** CCK-8 essay for BMSCs co-cultured with leonurine for 3 days illustrated increases in viability with leonurine-treated BMSCs. **(B)** Assessment of cell cycle phase distribution of leonurine –treated BMSCs. **(C)** Quantitative analysis of cell cycle phase distribution. No significant differences were measured between groups. **(D)** Distribution of apoptotic BMSCs observed under flow cytometry (FITC-Annexin V apoptotic detection assay). **(E)** Ratio of apoptotic BMSCs cultured in 0-10μM leonurine for 6 days. No significant differences were measured between groups (*p < 0.05, **p < 0.01, ***p < 0.001).

To assess the cytotoxicity of leonurine and its effect on the proliferation of BMSCs, we conducted a series of CCK-8 cell viability assays, cell cycle distribution analyses, and apoptosis assays with flow cytometry. As shown in Figure 1A, our results demonstrated that no apparent cytotoxicity was observed in the leonurine-treated group after 72 h of coculture with BMSCs. In contrast, viability was significantly increased in the BMSCs treated with leonurine at a range of 2–100 μM, with a concentration of 10 μM resulting in a peak increase in viability followed by a gradual decrease at higher concentrations (40–100 μM). It reflects the fact that 10 μM leonurine is the lowest and most proper functional concentration on BMSCs.

The flow cytometric results of BMSCs cocultured with leonurine demonstrated no notable change in the distribution of any particular cell cycle stage (Figures 1B,C). We observed an effect only on cell proliferation. However, whether the cell cycle period was changed was unclear. Apoptosis assays after coculture for 3 days further confirmed that leonurine did not have apparent toxicity and did not cause cell apoptosis (including early apoptosis and late apoptosis) (Figures 1D,E). These data proved the security of leonurine on BMSCs, and the lowest functional concentration is 10 μM.

### Leonurine Contributes to Osteoblast Differentiation

To investigate the effect of leonurine on osteoblast differentiation, we performed ALP staining and Alizarin red staining after 6 and 14 days of culture, respectively, as an early marker for osteoblastic differentiation and a late marker for calcium deposition in osteoblasts.

**FIGURE 2:**
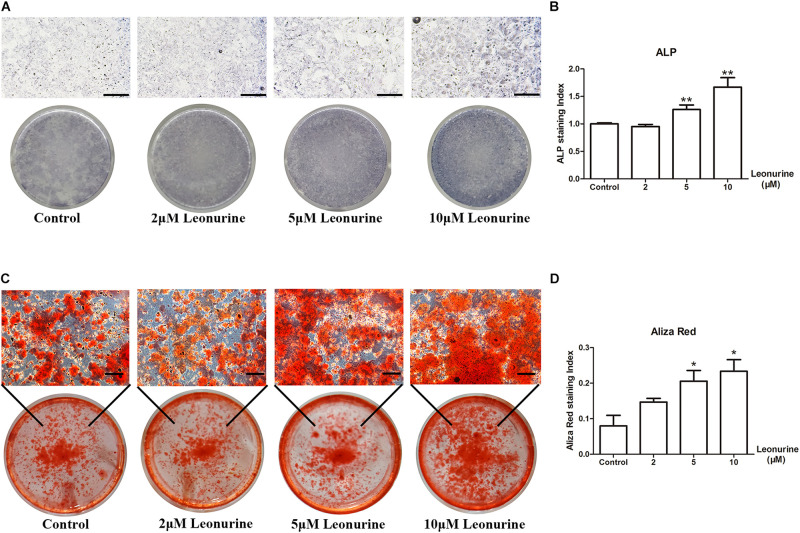
Effects of leonurine on osteoblast differentiation and mineralization of BMSCs. **(A)** ALP staining of leonurine-treated groups (0–10 μM) at day 6. **(B)** Quantitative analysis of ALP staining. Significant increases were recorded for 5 and 10 μM leonurine-treated groups. **(C)** Aliza red staining of leonurine-treated groups (0–10 μM) at day 14. **(D)** Quantitative analysis of ALP staining. Significant increases in mineralization were recorded for 5 and 10 μM leonurine-treated group (*p < 0.05, **p < 0.01, ***p < 0.001 vs. Control group). Scale bar = 500 μm.

As shown in Figure 2, leonurine contributed to osteoblastic differentiation in a dose-dependent manner, which was most apparent in the 10 μM leonurine-treated group (Figure 2A), with a significant increase at day 6 (Figure 2B). Alizarin red staining yielded comparable results after 14 days of culture, where a significant increase in mineralization was recorded for the 10 μM leonurine-treated BMSCs (Figures 2C,D). Combined, these results demonstrated the significant effect of 10 μM leonurine (compared to lower concentrations) in improving osteogenesis; we thereafter selected 10 μM leonurine as the concentration for subsequent analyses.

### Leonurine Promotes Osteogenic Differentiation via the Activation of Autophagy

**FIGURE 3:**
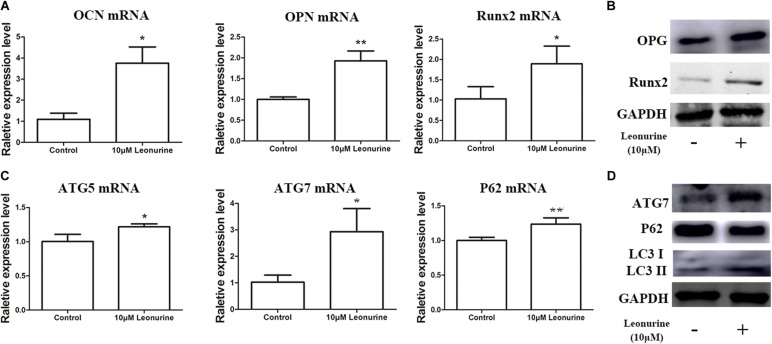
Effects of leonurine on osteogenic and autophagic activity in BMSCs. **(A)** qRT-PCR analysis of osteogenesis related genes *OCN*, *OPN*, and *Runx2* expression in BMSCs after 6 days of 10 μM leonurine treatment. Expression of all 3 mRNAs were significantly increased compared to control. **(B)** Western blot analysis of osteogenesis related proteins OPG and Runx2 in BMSCs after 6 days of 10μM leonurine treatment. Both proteins were significantly increased post-treatment. **(C)** Expression of autophagy related genes *ATG5*, *ATG7*, and *P62* in BMSCs after 6 days of 10 μM leonurine treatment. Expression of all 3 mRNAs were significantly increased compared to control. **(D)** Expression of autophagy related proteins ATG7, P62, LC3 I, and LC3 II in BMSCs after 6 days of 10 μM leonurine treatment. Both proteins were significantly increased post-treatment (*p < 0.05, **p < 0.01, ***p < 0.001 vs. Control group).

To determine the contribution of leonurine to osteogenesis, we performed qRT-PCR and WB analysis to further quantify the results obtained via ALP and Alizarin red staining. As shown in Figure 3, the addition of 10 μM leonurine improved the expression of osteogenesis-related markers (OCN, OPN, and Runx2) at both the mRNA and protein levels (Figures 3A,B).

Owing to the significant correlation present between autophagic activity and osteogenesis, autophagy-related mRNA and proteins, including ATG7, P62, and LC3 I/II, were subsequently quantified to determine whether leonurine could significantly modulate autophagy in BMSCs. Both qRT-PCR and WB analyses demonstrated that autophagy was enhanced in the leonurine-treated BMSCs compared to the untreated control cells (Figures 3C,D). This finding indicates that leonurine activated autophagy while accelerating the osteogenic process, demonstrating a strong relationship between them.

**FIGURE 4:**
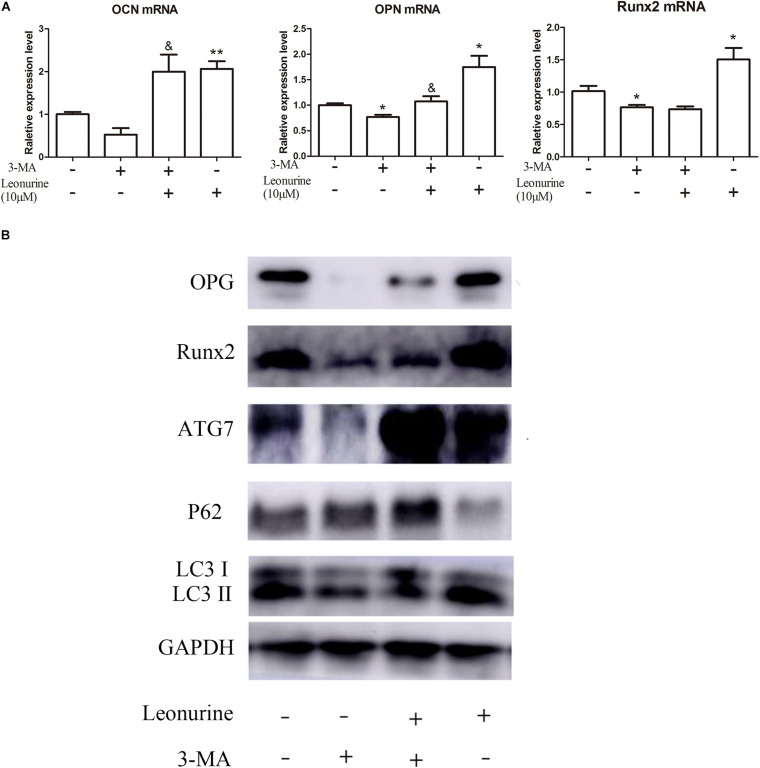
Modulation of osteogenic and autophagic activity by leonurine in autophagy-inhibited BMSCs **(A)** qRT-PCR analysis of osteogenesis related genes *OCN*, *OPN*, and *Runx2* in BMSCs treated with 10 μM leonurine, under the presence of the autophagy inhibitor 3-MA at 2 mM concentration. Leonurine partially rescued the decrease in *OCN* and *OPN* expression under the presence of 3-MA. (*p < 0.05, **p < 0.01, ***p < 0.001 vs. Control group, ^&^*p* < 0.05, ^&&^*p* < 0.01, ^&⁣&&^*p* < 0.001 vs. 3-MA group) **(B)** Western blot analysis for autophagy and osteogenesis related protein change in BMSCs treated with 10μM leonurine, under the presence of 3-MA at 2 mM concentration. The decrease in expression of both osteogenesis and autophagy related proteins due to 3-MA was partially rescued by the addition of 10 μM leonurine.

To further investigate the correlation between leonurine-induced activation of autophagy and its contribution to osteogenesis, we applied the autophagic inhibitor 3-MA with or without coculture treatment with leonurine. qRT-PCR and WB analyses both demonstrated significant inhibition of osteogenic activity with 3-MA treatment (Figures 4A,B), indicating a decrease in osteogenesis via the inhibition of autophagy in BMSCs; concurrently, autophagy was partly rescued with leonurine supplemented with 3-MA (Figures 4A,B).

**FIGURE 5:**
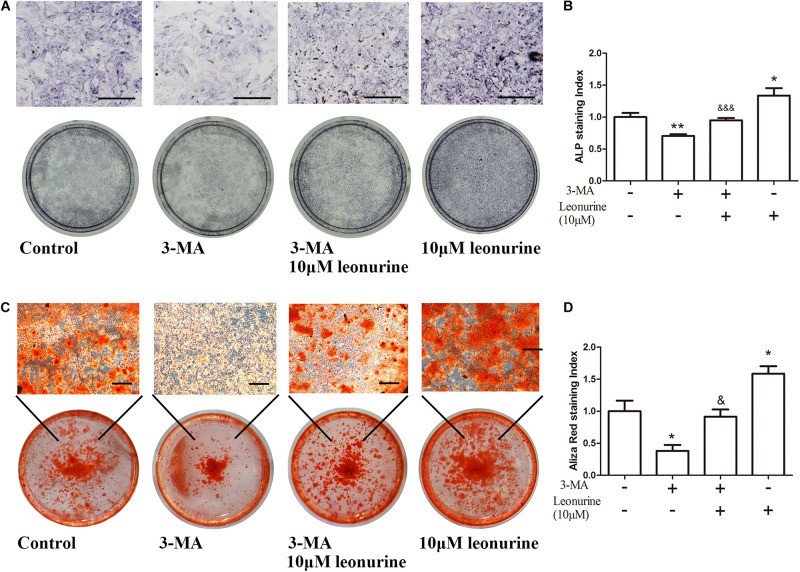
Effects of leonurine on osteoblast differentiation and mineralization of autophagy-inhibited BMSCs. **(A)** ALP staining of 0–10 μM leonurine treated BMSCs at day 6, with or without 2 mM 3-MA for autophagy inhibition. 10 μM leonurine partially reversed the inhibition of osteoclastic differentiation by 3-MA. **(B)** Quantitative analysis of ALP staining. **(C)** Alizarin red staining of 0–10 μM leonurine treated BMSCs at day 14, with or without 2 mM 3-MA for autophagy inhibition. 10μM leonurine partially reversed the inhibition of mineralization by 3-MA. **(D)** Quantitative analysis of Aliza red staining (Scale bar is 200μM) (*p < 0.05, **p < 0.01, ***p < 0.001 vs. Control group, ^&^*p* < 0.05, ^&&^*p* < 0.01, ^&⁣&&^*p* < 0.001 vs. 3-MA group).

As additional evidence of the autophagic and osteogenic modulation of leonurine, significant inhibition of osteoblastic differentiation and mineralization by 3-MA was shown by ALP assays and Alizarin red staining after 6 and 14 days of culture, respectively, this phenomenon was reversed after the addition of 10 μM leonurine (Figures 5A,C). The quantitative analysis is shown in Figures 5B,D.

As shown by this evidence, autophagic deficiency caused by 3-MA significantly decreased the osteogenic differentiation process, and these results support our hypothesis that leonurine can activate autophagy to contribute to osteogenesis.

### Leonurine Can Activate Autophagy by Inhibiting the PI3K/Akt/mTOR Pathway

**FIGURE 6:**
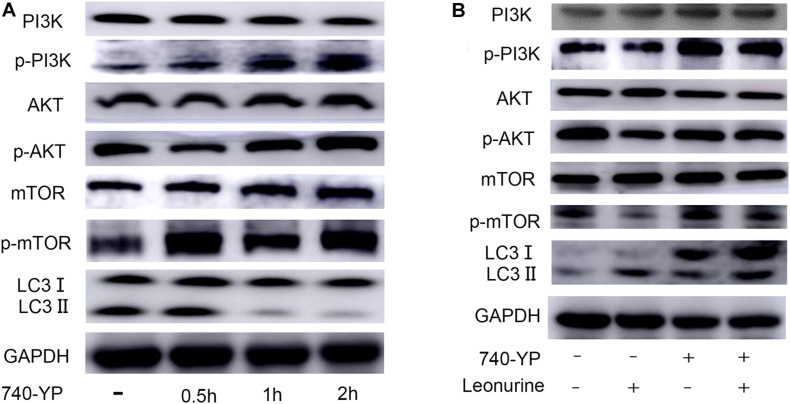
Modulation of PI3K-AKT-mTOR pathway by leonurine. **(A)** Western blot analysis on the addition of the PI3K activator 740-YP in BMSCs. 740-YP at a concentration of 2 μM increased expression of all downstream proteins in the PI3K-AKT-mTOR pathway. **(B)** Western blot analysis of the effect of leonurine on PI3K-AKT-mTOR pathway in BMSCs at 2 h. Expression of downstream proteins in PI3K-AKT-mTOR pathway in BMSCs treated with or without 2 μM 740-YP was negatively regulated by the addition of 10 μM leonurine.

Considering the importance of the PI3K pathway involved in autophagy and leonurine previous report, we investigated the influence of leonurine on the PI3K pathway to ascertain its possible effect in modulating autophagy. Our results indicated that the PI3K activator activates PI3K within 2 h, which is accompanied by the inhibition of autophagy (Figure 6A). Leonurine inhibited PI3K/Akt/mTOR activity with downregulation of phosphorylated PI3K/AKT/mTOR and exerted a negative effect against the PI3K activator 740-Y-P (Figure 6B). This evidence demonstrated that leonurine potentially activates autophagy via inhibition of the PI3K/Akt/mTOR pathway.

## Discussion

In this study, we demonstrated that leonurine, a natural compound derived from *Leonurus*, contributes to autophagy to improve BMSC differentiation without apparent cytotoxicity. Next, we further investigated its mechanism of autophagic activation by inhibiting the PI3K/Akt/mTOR pathway. Our results provide a rationale for developing leonurine as a new medicine for treatment of osteoporosis.

Many antiosteoporotic medicines result in various unavoidable side effects. In contrast, substantial evidence indicates a lowered risk of adverse events in treating osteoporosis with traditional Chinese medicine. Clinical studies have demonstrated that certain traditional Chinese medicines and their compounds can not only decrease bone resorption but also contribute to bone formation through estrogen-like effects, antioxidant activity, and modulation of bone metabolism (Zhang et al., 2016). For example, berberine promotes the differentiation of osteoblasts of BMSCs by stabilizing Runx2/Osterix through the increased activation of PKA signaling (Han et al., 2017). Kaempferol inhibits glucocorticoid-induced bone loss by promoting osteoblast survival through activation of the ERK pathway (Adhikary et al., 2018). Alisol-B suppresses RANKL-induced osteoclast formation to prevent bone loss through suppression of RANKL-mediated JNK pathway activation (Lee et al., 2010). Regarding the activity and effects of leonurine, it has been demonstrated that leonurine exhibits a protective function against cardiovascular disease, stroke, and nervous system disease by suppressing oxidative stress and chronic inflammation (Liu et al., 2013; Li et al., 2017). A recent study confirmed that leonurine has an antiosteoporotic effect on osteoclasts by inhibiting the PI3K-AKT and NF-κB signaling pathways (Yuan et al., 2015). To further elucidate this topic, we investigated the antiosteoporotic effect of leonurine on BMSC functional recovery. Our results illustrated that leonurine promoted the proliferation of BMSCs at appropriate concentrations. Concurrently, leonurine promoted bone mineralization, as shown by ALP staining and Alizarin red staining, along with upregulation of both osteogenic genes and proteins at a concentration of 10 μM. Combined, these results primarily indicated that leonurine can contribute to BMSC proliferation and differentiation, improving mineralization.

Osteoporosis associated with aging is characterized by consistent changes in bone metabolism with suppression of bone formation as well as increased bone resorption, both of which are associated with abnormal autophagic activity in osteoblasts (Bianco et al., 2011). Currently, there is substantial evidence to illustrate that autophagy can strongly contribute to osteoblast survival, regulate osteoblast differentiation, maintain bone mass, and improve bone strength : in detail, bone marrow-derived mesenchymal stem cells have been regarded as the main contributors of osteoblasts to bone formation (Noort et al., 2002). With increasing age, bone marrow-derived mesenchymal stem cells tend to lose their self-renewal capacity and are directed toward adipogenic differentiation instead of osteogenesis; this phenomenon contributes significantly to bone loss and lipid accumulation in bone marrow (Moerman et al., 2004). Related studies have revealed that autophagy is a necessary factor for maintaining stemness and differentiation and that defective autophagy contributes to a decline in both cell count and cellular functions (Garcia-Prat et al., 2016).

The decline in autophagic activity is strongly correlated with osteoporosis, in which the inhibition of autophagy leads to increased cell apoptosis and activation of autophagy contributes to increased cell viability (Yang et al., 2014). Previous studies have demonstrated that knockout of the autophagy-related genes *BECN-1*, *ATG7*, and *LC3* results in defective bone mineralization; concurrently, enhancing autophagy helps BMSCs retain their multipotency (Sargolzaeiaval et al., 2018). Prior research has examined the contribution of leonurine to the regulation of autophagy. In this study, we demonstrated that leonurine successfully activated autophagy in BMSCs and increased the expression of autophagy-related proteins during the BMSC differentiation stage. Following leonurine administration, the impaired osteogenic differentiation in BMSCs induced by the autophagic inhibitor 3-MA was partially recovered. Combined, this evidence indicates that leonurine could regulate BMSC function by activating autophagy.

Substantial evidence has confirmed the relationship between dysregulated autophagy and osteoporosis *in vitro* and *in vivo* (Liu et al., 2018). Based on these studies, autophagic activation was shown to contribute to osteogenesis and serves as a promising target in treating osteoporosis. Many studies have investigated autophagy-related pathways. Among them, mTOR-related pathways, including PI3K/Akt/mTOR, are highly involved in the regulation of cell autophagy (Gao et al., 2016), and evidence identifies the mTOR signaling pathway as a modulatory factor in mediating human osteoblastic differentiation. Deletion of Raptor, an essential component of the mTORC1 gene, in osteocytes did not affect bone development and growth but led to increased trabecular bone mass (Liu et al., 2018). Relevant studies have indicated that suppressing the phosphorylation of mTOR leads to the activation of autophagy, concurrently eliciting antiapoptotic effects on BMSCs and osteoblasts (Yang et al., 2019). Furthermore, the latest research indicates that suppression of the PI3K/AKT/mTOR pathway is a protective factor in glucocorticoid-induced osteoporosis (Wang et al., 2020).

Recent research has shown that leonurine can inhibit RANKL-mediated osteoclastogenesis, reducing the loss of bone volume caused by estrogen deficiency, and evidence of its antiosteoporotic function via upregulated osteogenesis in BMSCs was presented. Our overall results support the hypothesis that leonurine promotes BMSC osteoblastic differentiation via its main action of autophagic activation through PI3K/Akt/mTOR pathway inhibition. Previous literature has demonstrated the successful modulation of the PI3K/Akt/mTOR pathway by leonurine in various diseases (Loh et al., 2010; Yuan et al., 2015). In another study, leonurine inhibited PI3K/Akt/mTOR pathway activation, and the results strongly indicated direct binding to the PI3K protein in chondrocyte cells. Therefore, there is a strong relationship between PI3K/Akt/mTOR and autophagy. We first clarified that leonurine induced inhibition of the PI3K pathway, which has a direct relationship with autophagy. Comparable results were found in our research, in which PI3K phosphorylation was inhibited after leonurine treatment. Concurrently, our results suggested that leonurine induces a negative effect on the PI3K/AKT/mTOR pathway, acting as a direct antagonist of the *PI3K*/AKT activator (740Y-P)-dependent signaling pathway in this study. This finding contributes to our understanding of the mechanism by which leonurine harnesses autophagic activity to stimulate osteogenesis. However, a majority of other research on the leonurine-involved pathway has mainly focused on the NF-κB pathway, which has a strong relationship with inflammation (Hoesel and Schmid, 2013). Osteoporosis-related research on leonurine reported that leonurine impedes osteoclasts differentiation by inhibition of PI3K/AKT and NF-κB pathway (Yuan et al., 2015). We combined the evidence of direct combination to PI3K protein to further test leonurine mechanism of PI3K/Akt/mTOR pathway regulation in osteoblasts. This compensates the research on anti-osteoporosis mechanism from another aspect. However, our research did not examine NF-κB pathway function in autophagy, and this crosstalk with the PI3K pathway should further be taken into consideration for analysis of the mechanism of leonurine in a future study.

In conclusion, our research suggests the possible use of leonurine in activating BMSC autophagy to treat osteoporosis. Leonurine inhibits the PI3K/Akt/mTOR pathway to activate autophagy, subsequently contributing to osteoblast differentiation. These results strongly suggest that leonurine is a candidate medicine for potential studies in developing new therapies for osteoporosis.

## Data Availability Statement

The original contributions presented in the study are included in the article, further inquiries can be directed to the corresponding authors.

## Author Contributions

SQ designed the experiment and was responsible for the reviewers’ suggestion. RW helped designed the experiments and checked uploaded data and rearranged the documents. YX and RW funded the experiments. BZ carried out the experiment and wrote the manuscript. QP, RZ, and GS were assistant of experiments. DW and EP helped the language edition. All authors contributed to the article and approved the submitted version.

## Conflict of Interest

The authors declare that the research was conducted in the absence of any commercial or financial relationships that could be construed as a potential conflict of interest.
